# TV parenting practices: is the same scale appropriate for parents of children of different ages?

**DOI:** 10.1186/1479-5868-10-41

**Published:** 2013-04-02

**Authors:** Tzu-An Chen, Teresia M O’Connor, Sheryl O Hughes, Leslie Frankel, Janice Baranowski, Jason A Mendoza, Debbe Thompson, Tom Baranowski

**Affiliations:** 1 USDA/ARS Children’s Nutrition Research Center, Baylor College of Medicine, 1100 Bates Street, Rm. 4012, Houston, TX, USA

**Keywords:** TV, Parenting practices, Multidimensional, Item response modeling, Differential item functioning

## Abstract

**Purposes:**

Use multidimensional polytomous item response modeling (MPIRM) to evaluate the psychometric properties of a television (TV) parenting practices (PP) instrument. Perform differential item functioning (DIF) analysis to test whether item parameter estimates differed across education, language, or age groups.

**Methods:**

Secondary analyses of data from three studies that included 358 children between the ages of 3 and 12 years old in Houston, Texas. TV PP included 15 items with three subscales: social co-viewing, instructive parental mediation, and restrictive parenting. The multidimensional partial credit model was used to assess the performance. DIF was used to investigate the differences in psychometric properties across subgroups.

**Results:**

Classical test theory analyses revealed acceptable internal consistency reliability (Cronbach’s α: 0.72 to 0.83). More items displaying significant DIF were found across children’s age groups than parental education or language groups. A Wright map revealed that items covered only a restricted range of the distribution, at the easier to respond end of the trait.

**Conclusions:**

TV PP scales functioned differently on the basis of parental education, parental language, and child age, with the highest DIF among the latter. Additional research is needed to modify the scales to minimize these moderating influences. Some items may be age specific.

## Introduction

Television (TV) viewing increased among youth in the United States [1], and is considered a cause of childhood obesity [2-5]. Parenting practices to reduce children’s TV viewing may be important for preventing child obesity. Parenting practices (PP) are behaviors parents use to influence their child’s behaviors [6-8]. Limited psychometric analyses have been reported on TV PP scales with all having employed only classical test theory (CTT) [9]. CTT, however, is sample-dependent. In contrast, item response modeling (IRM) provides model-based measurements: trait level estimates obtained as a function of participants’ responses and properties of the administered items [10,11]. For example, the participants’ estimated trait level of TV PP depends both on a person’s response to these items and the items’ parameters.

Valid measures are needed both to understand how PP influence child behaviors and to measure mediating variables in parenting change interventions. PP that influence child TV viewing may differ depending on parental education level, child’s age or parent’s understanding of items that may differ by language [12-14]. Such differences could pose serious problems for validity by making it difficult to compare parameter estimates across these variables or across studies. Multidimensional polytomous item response modeling (MPIRM) enables differential item functioning (DIF) analysis [15] for multidimensional scales.

The aim of this study was to use MPIRM and DIF to examine the item and person characteristics of TV PP scales across education, language, and age groups.

## Methods

### Participants

Children (n = 358) between 3 to 12 years old (yo) in Houston, Texas, were included in the present analyses and the data were assembled from three studies: a physical activity intervention using Wii Active Video Games (Wii, n = 78) [16], a first line obesity treatment intervention Helping HAND (Healthy Activity and Nutrition Directions) (HH, n = 40) [17], and one cross-sectional study, Niños Activos (NA, n = 240). The Wii study recruited 84 children from multiple sources to participate in a 13-week exergame intervention in 2010. The inclusionary criteria targeted children 9–12 yo, whose BMI were within the 50-99th percentile range. Details have been reported elsewhere [16]. HH recruited 40 5–8 yo children whose BMI were within 85–99th percentile range to participate in an obesity treatment study in pediatric primary care. Details have been reported elsewhere [17]. Niños Activos recruited 240 3–5 yo Hispanic children from Houston, TX with no restrictions on BMI to participate in a study assessing influences on child PA. TV PP was assessed at baseline in all studies.

The Institutional Review Board of Baylor College of Medicine approved all three study protocols. Signed informed consent and assent were obtained from each parent and child.

### Instrument

All parents self-completed in English or Spanish a TV PP questionnaire [9] which was originally developed to assess the TV mediation styles of 519 Dutch parents of children 5–12 years old. In the original study, this scale contained 15 items distributed across 3 subscales: restrictive mediation (5 items, α = 0.79), instructive mediation (5 items, α = 0.79) and social co-viewing (5 items, α = 0.80) [9]. Restrictive mediation was defined as the parent determining the duration of TV viewing and specifying appropriate programs; instructive mediation was the parent explaining the meaning of TV programs and the acceptability of characters’ behaviors; and social co-viewing was a parent watching TV together with his/her child [17-19].

Items in the Wii and HH studies featured the same four response options as in the original studies (*Never, Rarely, Sometimes, and Often*). Items in the NA study featured five response options (*Never, Rarely, Sometimes, Often and Always*). To facilitate analyses, category response curves (CRCs) were depicted on the NA sample to determine the collapse of response categories. Parents provided demographic information in all three studies at baseline.

### Analyses

#### Classical test theory

Item difficulty (mean) and item discrimination (corrected item-total correlations, CITC) were first assessed for the TV PP scales, and Cronbach’s alpha assessed internal consistency reliability. Criteria for acceptable CITC and internal consistency reliability were defined as greater than 0.30 and 0.70, respectively [20]. All CTT analyses were conducted using Statistical Analysis Systems [21].

#### Item response modeling (IRM)

The primary assumption of IRM, unidimensionality, was tested using exploratory factor analysis in SPSS [22] for each subscale. Unidimensionality was satisfied if the scree plots showed one dominant factor, the solution explained at least 20% of variance for the first factor, and the factor loadings were >0.30 [23]. An IRM model which best explained the data structure was selected after unidimensionality was confirmed in each subscale.

Polytomous IRM models were used because the TV PP items presented multiple response possibilities [24,25]. Polytomous IRM modeled the probability of endorsing one response category over another, referred to as a *threshold parameter,* indicating the probability of responding at or above a given category. For an item with four response options (e.g., *never*, *rarely*, *sometimes*, and *often*), three thresholds exist (1) from “*never*” to “*rarely*”, (2) from “*rarely*” to “*sometimes*”, and (3) from “*sometimes*” to “*often*”. The item threshold locations were determined along the latent trait continuum. The latent trait estimates from IRM can be related to the raw scores of the TVPP scale using non-linear transformation.

Category response curves (CRC) show the probability of a response in a particular category for a given trait level. The number of CRCs equals the number of response options. In this study, every item has four CRCs, and each CRC shows the probability of endorsing the particular response at different levels of the latent trait. For example, CRCs for response option *“rarely”* show at what latent trait level participants will be more likely to endorse *“rarely”* than the other three response categories. The sum of the response probabilities equals 1.0 at any location along the underlying trait continuum. CRCs can also be used to identify the most likely response at various levels of a latent trait.

Item-person maps, often called Wright maps (with units referred to as *log odds*), depicted the distributions of scale items with that of the respondents along the latent trait on the same scale. The dashed vertical line presents the latent trait in logits which were specified on the far left of the map. A logit of 0 in this map implies a moderate amount of latent trait. The location of thresholds in a Wright map shows the point at which the probability of the scores below *k* equals the probability of the scores *k* and above. For example, the location of Threshold 1 shows the amount of latent trait of the corresponding sub-scale, e.g. restrictive TVPP, a person must possess if there is a 0.5 probability of selecting *“rarely”* over *“never”*. Large gaps along the difficulties continuum imply that additional items will help distinguish within that particular range of difficulty. Since the TV PP instrument contained three sub-scales, two multidimensional polytomous models were considered: partial credit (PCM) [26], and rating scale models (RSM) [27,28]. RSM is a special case of the PCM where the response scale is fixed for all items, i.e., the response threshold parameters are assumed to be identical across items. The relative fit of RSM and PCM was evaluated by considering the deviance difference, where *df* was equal to the difference in the number of estimated parameters between the two models.

Item fit was assessed using information-weighted fit statistic (infit) and outlier-sensitive fit statistic (outfit) mean square index (MNSQ) which have possible ranges from zero to infinity. Infit MNSQ is based on information-weighted sum of squared standardized residuals; outfit MNSQ is a sum of squared standardized residuals [29]. An infit or outfit MNSQ value of one indicates the observed variance equals the expected variance. MNSQ values greater than, or smaller than, one indicate the observed variance is greater, or smaller, than the expected, respectively. Infit or outfit MNSQ values greater than 1.3 indicate poor item fit (for n < 500 [30,31] with significant *t*-values. Concerning thresholds, outfit MNSQ values greater than 2.0 indicate misfit, identifying candidates for collapsing with a neighboring category [29,32].

#### Differential item functioning (DIF)

Participants with the same underlying trait level, but from different groups, may have different probabilities of endorsing an item. DIF was assessed by an item-by-group interaction term [33,34], with a significant chi-square for the interaction term indicating DIF. Items display DIF if the ratio of the item-by-group parameter estimates to the corresponding standard error exceeds 1.96. A finding of DIF by gender means that a male and a female with the same latent trait level responded differently to an item, suggesting that respondents’ interpretation of the item differed for males and females.

The magnitude of DIF was determined by examining the differences of the item-by-group interaction parameter estimates. Because the parameters were constrained to be zero, if only two groups were considered the magnitude of DIF difference was twice the estimate of the first focal group. If comparison was made among three or more groups, the magnitude of DIF was the difference of the interaction term estimate of the corresponding groups. Items were placed into one of three significant DIF categories depending on the effect size: small (difference < 0.426), intermediate (0.426 < difference < 0.638), and large (difference > 0.638) DIF [35,36]. ACER (Australian Council for Educational Research) ConQuest [37] was used for all IRM analyses.

## Results

### Descriptive statistics

Participant demographic characteristics are shown in Table 1 by source. Parental education level was almost evenly distributed across the three studies with 50.7% of all participants reporting a high school education or less education. Because these three original studies recruited kids at different age ranges, percentages by age group in the combined sample were proportional to the original study’s sample size; 57.8% completed the English version. The majority of participants were Hispanic (79.1%).

**Table 1:** Demographic characteristics of respondents

	**Wii**		**HH**		**NA**		**Total**	
	**n**	**%**	**n**	**%**	**n**	**%**	**n**	**%**
**Education**								
High School or Less	15	19.23	27	67.50	139	58.16	181	50.70
Above High School	63	80.77	13	32.50	100	41.84	176	49.30
**Language**								
English	78	100.00	17	42.50	112	46.67	207	57.82
Spanish	0	0.00	23	57.50	128	53.33	151	42.18
**Ethnicity**								
Hispanic	10	12.8	33	82.50	240	100.00	283	79.05
Non-Hispanic	68	87.2	7	17.50	0	0.00	75	20.95
**Age**								
3-5 years old	0	0.00	0	0.00	240	100.00	240	67.04
5-8 years old	0	0.00	40	100.00	0	0.00	40	11.17
9-12 years old	78	100.00	0	0.00	0	0.00	78	21.79

Note. Wii = children participating in the Wii exergame study [16]; HH = children participating in the Helping Hand study [17]; NA = children participating in the Niños Activos study.

### Category response curves (CRCs)

**Figure 1:**
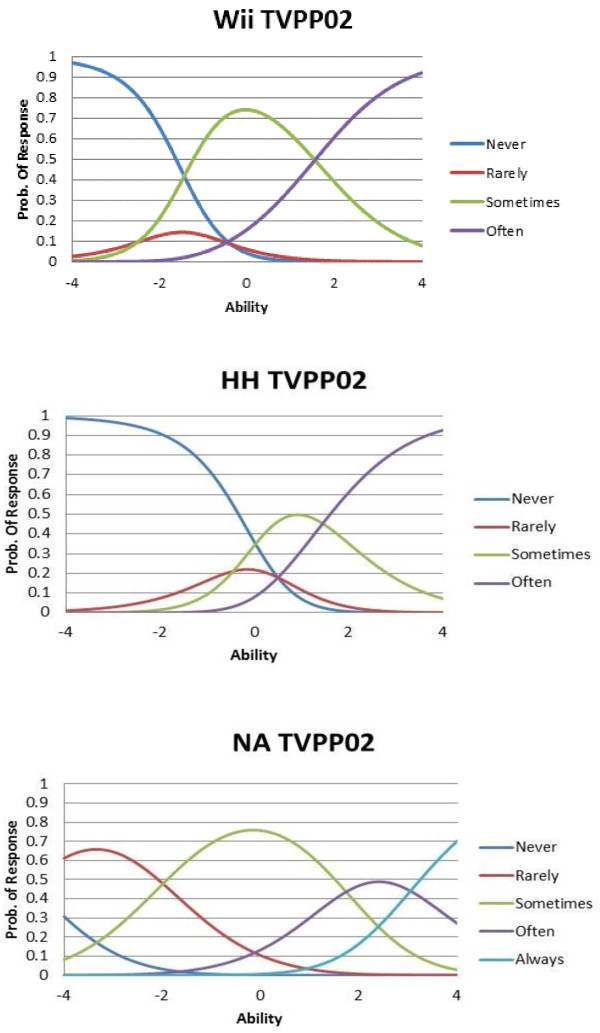
Category Response Curves for Item 2: “How often do you explain what something on TV really means?”.

The CRCs for the response category “*often”* mostly never peaked for the NA sample across the 15 items, indicating that *“often”* never had the highest probability of being selected for most items. Therefore, the response categories *“Often”* and *“Always”* were collapsed in the NA sample. Figure 1 shows CRCs for item 2 across the three original samples (Wii, HH and NA). The curve for “*rarely*” never peaked in two of the samples, indicating that respondents were unlikely to choose “*rarely*”. CRCs revealed that respondents did not use all response categories (usually only 3), and response category use differed by sample. (CRCs for the remaining items are available upon request).

### Classical test theory

The percentage of variance explained by the one-factor solution was 60%, 60% and 48% for social co-viewing, instructive mediation and restrictive subscales, respectively. The scree plots revealed one dominant factor and factor loadings were >0.30 for all three subscales.

Item difficulty (item means) ranged from 3.16 (SD = 0.49) to 3.67 (SD = 0.62), indicating that on average respondents reported frequently performing the PP. Internal consistencies were good for social co-viewing (α = 0.83); and instructive parental mediation, (α = 0.83); and adequate for restrictive mediation (α = 0.72). CITCs acceptably ranged from 0.41 to 0.70.

### IRM model fit

The chi-square (*χ*^2^) deviance statistic was calculated by considering differences in model deviances (RSM: 8749.63; PCM: 8485.99) and differences in numbers of parameters (RSM: 23; PCM: 51) for the nested models. The chi-square test of the deviance difference showed RSM significantly reduced model fit (Δ deviance = 263.64, Δ *df* = 28, p < 0.0001); thus, further analyses employed multidimensional PCM.

### Item fit

Item difficulties are summarized in Table 2. Assuming a multidimensional PCM, only one item (item 2) exceeded the criterion guideline (> 1.3). Item 14 was flagged as the only misfit item when taking into account the difference in parental education level (infit/outfit MNSQ = 1.37), in language (infit/outfit MNSQ = 1.32), or in child’s age (infit MNSQ = 1.36; outfit MNSQ = 1.42). Misfit values were relatively small; therefore, the items were retained in the ensuing analyses.

**Table 2:** Item description, item difficulty, and misfit item(s)

					**Age**		
**Items**	**Item questions**	**All**	**Low edu.**	**English**	**3-5 yo**	**5-8 yo**	**9-12 yo**
	**Social Co-viewing **(Cronbach’s alpha = 0.83)						
6	How often do you laugh with your child about the things you see on TV?	-1.02	-0.16	0.00	0.06	-0.29	0.23
1	How often do you watch TV together because you both like a program?	0.04	0.06	-0.10	-0.11	0.17	-0.06
12	How often do you watch TV together because of a common interest in a program?	0.14	0.02^b^	0.15^c^	0.23^d^	-0.11^d^	-0.12^d^
14	How often do you watch TV together just for fun?	0.20	0.24	-0.21	0.08	0.08	-0.16
5	How often do you watch your favorite program together?	0.64	-0.15	0.15	-0.26	0.15	0.11
	**Instructive Mediation **(Cronbach’s alpha = 0.83)						
2	How often do you explain what something on TV really means?	-0.59^a^	0.33	-0.30	0.21	0.47	-0.68
4	How often do you try to help your child understand what she/he sees on TV?	-0.40	-0.04	-0.05	-0.21	0.01	0.19
8	How often do you point out why some things actors do are bad?	0.08	-0.14	0.17	0.14	-0.36	0.22
10	How often do you point out why some things actors do are good?	0.18	-0.02	0.03	0.06	-0.06	0.00
13	How often do you explain the motives of TV characters?	0.72	-0.13	0.16	-0.20	-0.07	0.27
	**Restrictive **(Cronbach’s alpha = 0.72)						
15	How often do you forbid your child to watch certain programs?	-0.38	0.04	-0.06	0.01	-0.18	0.17
9	How often do you tell your child to turn off the TV when he/she is watching an unsuitable program?	-0.25	-0.06	0.13	0.10	0.01	-0.10
3	How often do you specify in advance the programs that may be watched?	-0.07	0.02	0.03	0.16	-0.26	0.10
11	How often do you restrict the amount of TV your child can watch?	0.09	-0.07	0.07	-0.25	0.20	0.05
7	How often do you set specific viewing hours for your child?	0.62	0.07	-0.17	-0.01	0.23	-0.22

Note. ^a^Misfit item (Item 2) Outfit MNSQ = 1.34; ^b^ Misfit item (Item 14) Infit/Outfit MNSQ = 1.37; ^c ^Misfit item (Item 14) Infit/Outfit MNSQ = 1.32;^d^Misfit item (Item 14) Infit MNSQ = 1.36; Outfit MNSQ = 1.42.

Item difficulties for High edu. and Spanish were not shown here since the estimates were constrained to be zero, the item difficulty will have opposite sign if only two groups were considered. For example, item difficulty of item 1 for High edu. is -0.06.

### Item-person fit Wright map

**Figure 2:**
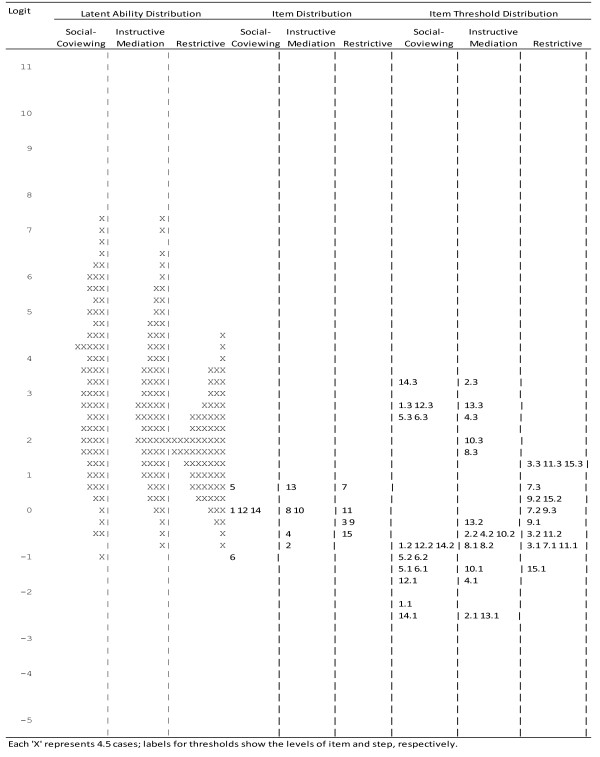
Wright Map of TV PP Scale (n=358).

Figure 2 presents the multidimensional PCM item-person maps. Person, item and threshold estimates were placed on the same map where “*x*” on the left side represented the trait estimates of a person with the parent scoring in the highest TV PP range placed at the top of the figure. Item and threshold difficulties were presented on the right side, with the more difficult response items and thresholds at the top. The range of item difficulties was narrow (logits ranged from -1.02 to 0.72); the distribution of item difficulties did not match that of individuals for each dimension. In each subscale category, most parents found it easy to endorse these items. Many items’ (1, 2, 4–6, 10 and 12–15) first step threshold did not coincide with participants at the lower end of TV PP.

### Differential item functioning (DIF)

Item difficulty differences between demographic groups are presented in Table 3. One, five and nine items exhibited significant DIF between educational level, language, and child’s age groups, respectively (Table 3). Only item 2 had significant DIF by educational level at 0.67, a large DIF effect: it was easier for parents with higher education level to endorse item 2. Moderate DIF was detected for item 2 and small DIF for items 5, 7, 8, and 9 by use of the English or Spanish version. The Spanish version users found it somewhat easier to endorse items 5, 8, and 9, but more difficult to endorse items 2 and 7. Medium DIF was detected for items 8 and 11 between children of ages 3–5 years and children of ages 5–8. Large DIF was indicated for item 2 between children 3–5 yo and children 9–12 yo, and between children 5–8 yo and children 9–12 yo. It was easier for parents with older children to endorse items 6, 12, 2, and 8 and for parents with younger children to endorse items 5, 4, 7, and 11.

**Table 3:** Item description and estimates of DIF where significant

**Items**	**Item questions**	**CTT**		**IRM**				
		**Mean (SD)**	**CITC**	**Difficulty differences**				
				**Low edu.-High edu.**^**a**^	**English-Spanish**^**b**^	**3-5 yo -5-8 yo**^**c**^	**3-5 yo-9-12 yo**^**d**^	**5-8 yo -9-12 yo**^**e**^
	**Social Co-viewing **(Cronbach’s alpha = 0.83)							
1	How often do you watch TV together because you both like a program?	3.42 (0.67)	0.68					
5	How often do you watch your favorite program together?	3.35 (0.74)	0.7		0.3*	-0.41*	-0.37*	
6	How often do you laugh with your child about the things you see on TV?	3.67 (0.54)	0.62			0.35*		
12	How often do you watch TV together because of a common interest in a program?	3.41 (0.67)	0.67			0.34*		
14	How often do you watch TV together just for fun?	3.35 (0.67)	0.51					
	**Instructive Mediation **(Cronbach’s alpha = 0.83)							
2	How often do you explain what something on TV really means?	3.47 (0.63)	0.54	0.67***	-0.6**		0.88***	1.15***
4	How often do you try to help your child understand what she/he sees on TV?	3.53 (0.66)	0.58				-0.4*	
8	How often do you point out why some things actors do are bad?	3.53 (0.74)	0.65		0.34*	0.5**		
10	How often do you point out why some things actors do are good?	3.44 (0.74)	0.69					
13	How often do you explain the motives of TV characters?	3.19 (0.77)	0.7					
	**Restrictive **(Cronbach’s alpha = 0.72)							
3	How often do you specify in advance the programs that may be watched?	3.47 (0.76)	0.41			0.41*		-0.36*
7	How often do you set specific viewing hours for your child?	3.16 (0.96)	0.49		-0.33*	-0.24*		
9	How often do you tell your child to turn off the TV when he/she is watching an unsuitable program?	3.65 (0.74)	0.42		0.25*			
11	How often do you restrict the amount of TV your child can watch?	3.39 (0.78)	0.56			-0.46**	-0.3*	
15	How often do you forbid your child to watch certain programs?	3.51 (0.72)	0.54					

Note.*small effect (difference < 0.426); **moderate effect (0.426 < difference < 0.638); ***large effect (difference > 0.638).

^a^Positive numbers, easier for high education; Negative value, easier for low education; ^b^Positive numbers, easier for Spanish; Negative value, easier for English.

^c^Positive numbers, easier for 5–8 years old; Negative value, easier for 3–5 years old; ^d^Positive numbers, easier for 9–12 years old; ^e^Negative value, easier for 3–5 years old.

## Discussion

This is the first study to present an analysis using multidimensional PCM for a TV PP instrument. While CTT analyses indicated that the scales yielded generally acceptable (good or adequate) reliability, item characteristic curves revealed respondents used only 3 of 4 response categories. Thus, it appears appropriate to simplify response categories to 3 options in the future. The asymmetric distribution of items and item thresholds against individuals on the Wright map indicated the items and thresholds did not cover the more difficult to endorse end of each of the three latent variable dimensions. This suggests that items should be developed to cover the more difficult extreme end for each dimension.

DIF analyses indicated that some items did not behave the same way across subgroups. A large amount of DIF was identified for item 2 (i.e. “How often do you explain what something on TV really means?”) on the basis of education of parent and age of child; medium DIF was detected for item 2 on the language version, and for items 8 and 11 on children’s age. Parents with 3–5 yo kids tended to watch favorite programs together, and more likely restricted the amount of TV viewing than parents with older kids. Parents with older kids (9–12 yo) and with higher education level showed a higher degree of agreement with explaining to their child what something on TV really meant. Parents with 5–8 yo kids were more likely to specify in advance the programs that kids may watch than the other two age groups. Parents who used the English version tended to help their kids understand the meaning of something on TV and set specific TV viewing time; while parents who completed the Spanish version tended to agree that they watched the favorite program together, pointed out why some things actors do are bad, and asked their child to turn off the TV when he/she was viewing an unsuitable program.

DIF by age group presents distinct issues. While the usual prescription for eliminating DIF is to rewrite items to enhance the clarity of meaning [38,39], it may be that these items are reasonably clear and just not equally applicable across all ages of children. This suggests that responses for such scales can only be analyzed within rather narrow age groupings. The optimal age groupings await determination in future studies with larger samples. These subscale items all reflect frequency of performance, which is common among behavioral indicators. There may be benefit in introducing a value or normative aspect to these items: should parents do each of these practices?

Several limitations exist. The response scales for the NA items (a 5-point rating scale) were different than those for items in the Wii and HH studies (4-point scale). Collapsing one of the response categories based on infrequent use was a reasonable accommodation, but having the same categories would have been preferred. The samples in the three studies reflected different inclusionary/exclusionary criteria and different recruitment procedures, with unknown effects on the findings. The Wii study with 9–12 yo did not include any participants using the Spanish version, therefore, DIF by age may confound language. Finally, the sample size was relatively small. While no clear standards for minimum sample size are available, Embretson and Reise [40] recommended using a sample of 500, and Reeve and Fayers [41] recommended at least 350. Finally, an interaction term was used to detect DIF. Further investigation should pursue other DIF-detection procedures (e.g., Mantel [42]; Shealy & Stout [43,44]).

## Conclusion

TVPP subscales demonstrated factorial validity and acceptable internal consistency reliability. The true latent variables demonstrated adequate fit to the data, but did not adequately cover the more difficult to respond end of each dimension; effectively used only these response categories; and showed differential item functioning, especially by age. While the scales can be cautiously used, further formative work is necessary.

## Abbreviations

CITC: Corrected item-total correlation; CRC: Category response curve; CTT: Classical test theory; HAND: Healthy Activity and Nutrition Directions; HH: Helping HAND; DIF: Differential item functioning; Infit: information-weighted fit statistic; IRM: Item response modeling; MNSQ: Mean square item fit index; MPIRM: Multidimensional polytomous item response modeling; NA: Niños Activos; Outfit: Outlier-sensitive fit statistic; PCM: Partial credit model; PP: Parenting practices; RSM: Rating scale model; TV: Television; TV PP: TV parenting practices; Wii: Wii Active Video Game; Yo: Years old.

## Competing interests

The authors declare that they have no competing interests.

## Authors’ contributions

TC participated in the design of the study, conducted the psychometrics analyses and drafted the manuscript. TB, TO, SH, LF, JB, JM, and DT helped conceive the study, and participated in its design and helped to critically edit the manuscript. All authors read and approved the final manuscript.
